# On event-based optical flow detection

**DOI:** 10.3389/fnins.2015.00137

**Published:** 2015-04-20

**Authors:** Tobias Brosch, Stephan Tschechne, Heiko Neumann

**Affiliations:** Faculty of Engineering and Computer Science, Institute of Neural Information Processing, Ulm UniversityUlm, Germany

**Keywords:** event-based sensor, motion detection, optical flow, address-event representation, motion integration, velocity representation, spatio-temporal receptive fields

## Abstract

Event-based sensing, i.e., the asynchronous detection of luminance changes, promises low-energy, high dynamic range, and sparse sensing. This stands in contrast to whole image frame-wise acquisition by standard cameras. Here, we systematically investigate the implications of event-based sensing in the context of visual motion, or flow, estimation. Starting from a common theoretical foundation, we discuss different principal approaches for optical flow detection ranging from gradient-based methods over plane-fitting to filter based methods and identify strengths and weaknesses of each class. Gradient-based methods for local motion integration are shown to suffer from the sparse encoding in address-event representations (AER). Approaches exploiting the local plane like structure of the event cloud, on the other hand, are shown to be well suited. Within this class, filter based approaches are shown to define a proper detection scheme which can also deal with the problem of representing multiple motions at a single location (motion transparency). A novel biologically inspired efficient motion detector is proposed, analyzed and experimentally validated. Furthermore, a stage of surround normalization is incorporated. Together with the filtering this defines a canonical circuit for motion feature detection. The theoretical analysis shows that such an integrated circuit reduces motion ambiguity in addition to decorrelating the representation of motion related activations.

## 1. Introduction

The initial stages of visual processing extract a vocabulary of relevant feature items related to a visual scene. Rays of light reach the observer's eye and are transformed to internal representations. This can be formalized as sampling the ambient optic array (Gibson, 1978, 1986). Formally, the plenoptic function *P*(θ, φ, λ, *t*, *V*_*x*_, *V*_*y*_, *V*_*z*_) describes the intensity of a light ray of wavelength λ passing through the center of the pupil of an idealized eye at every possible angle (θ, φ) located at the position (*V*_*x*_, *V*_*y*_, *V*_*z*_) at time *t* (Adelson and Bergen, 1991). As a simplification we assume a single stationary camera sensing a single narrow band of wavelengths in the electromagnetic spectrum on its image plane (*x*, *y*), reducing the plenoptic function to *P*_λ, *V*_*x*_, *V*_*y*_, *V*_*z*__(*x*, *y*, *t*) = *g*(*x*, *y*, *t*) (the spatio-temporal gray level function). Elemental measurements are necessary to access the plenoptic structures. Conventional frame-based cameras sample the optic array by reading out measurements of all light-sensitive pixels at a fixed rate. Since the temporal sampling rate is limited through reading all pixel values in a fixed time interval, fast local luminance changes are integrated over time and cannot be differentiated in the further processing. When no changes occur in the intensity function, redundant information is generated that is carried to the subsequent processing steps. Address-event representations (AER), on the other hand, originate from image sensors in which pixel operate at individual rates generating events based on local decisions to generate an output response, like in the mammalian retina (Mead, 1990; Liu and Delbruck, 2010).

**Figure 1:**
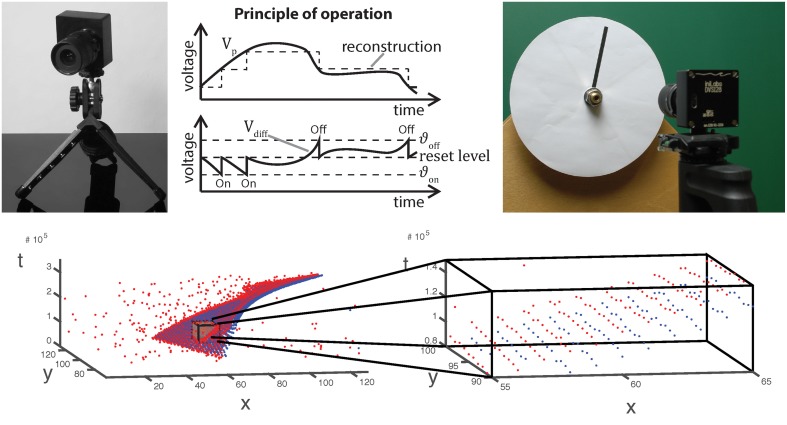
**Top from left to right:** Image, operating principle and stimulus generation of/for an asynchronous event sensor. Luminance changes exceeding a given threshold evoke ON and OFF events for positive and negative changes, respectively. The very low latency of the dynamic vision sensor (15 μs) requires analog stimulus generation as illustrated on the right. **Bottom:** Visualization of the spatio-temporal event cloud generated by the rotating stimulus in the upper right. A small volume has been zoomed in. As can be seen, only few events have been generated at a single location.

We will focus on silicon retinas that generate an AER, namely the dynamic vision sensor (DVS; Delbrück and Liu, 2004). Whenever the change in the log-luminance function exceeds a predefined threshold ϑ, events *e*_*k*_ ∈ {−1, 1} are generated at times *t*_*k*_ that emulate spike sequences of on- and off-contrast cells in the retina, respectively (Figure 1). We discuss what kind of information is accessible from the initial stages of event-based visual sensing and compare different approaches to estimate optical flow from the stream of on- and off-events visualized in Figure 1. We identify weaknesses, suggest improvements, propose a novel biologically inspired motion detector and conduct experiments to validate the theoretical predictions of flow estimation. The proposed detector is then further extended by incorporating an inhibitory pool of activation over a neighborhood in the space-time-feature domain that leads to contextual modulation and response normalization. Together with the initial filtering stage the scheme defines a canonical circuit model as suggested in Kouh and Poggio (2008); Carandini and Heeger (2012); Brosch and Neumann (2014a). This competitive mechanism is investigated from an information-theoretic point of view, shown to accomplish decorrelation, and linked to radial Gaussianization of the input response distribution (Lyu and Simoncelli, 2009b). Finally, we investigate whether motion transparency encoding (Snowden and Verstraten, 1999), i.e., the percept of two competing motions at a single location, like flocks of birds flying in front of passing clouds, can be supported.

## 2. Materials and methods

### 2.1. Theoretical aspects of event-based visual motion detection

#### 2.1.1. Nomenclature and principal problems

We describe the stream of events by the function

(1) $$e:{\mathbb{R}}^{2}\times \mathbb{R}\to \{-1,\;0,\;1\}$$

which is always zero except for tuples (*x*_*k*_, *y*_*k*_; *t*_*k*_) = (*p*_*k*_; *t*_*k*_) which define the location and time of an event *k* generated when the luminance function increases or decreases by a significant amount. In other words, the function that defines the event generation *e*(*p*_*k*_; *t*_*k*_) = *e*_*k*_, generates 1 if the log-luminance changed more than a threshold ϑ, i.e., an ON event, and −1 if it changed more than −ϑ, i.e., an OFF event. This sampling of the lightfield essentially represents the temporal derivative of the luminance function *g*

(2) $$\frac{d}{dt}g(p;t)=g_{t}(p;t)\approx \frac{\vartheta }{\Delta t}\sum_{{}_{k:\;t_{k}\;\in \;(t\;-\;\Delta t,t]}}e_{k},$$

with ϑ the sensitivity threshold of the event-based sensor.

**Figure 2:**
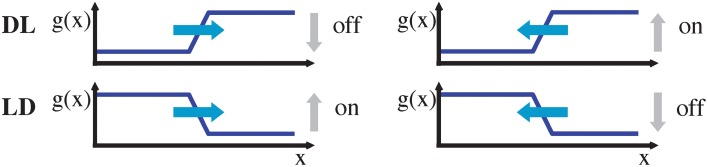
**Moving DL (dark-light) and LD (light-dark) edge, either to the left or to the right (denoted by blue arrows), have an associated temporal on/off signature**. Note that without knowledge about the edge type (DL vs. LD), an on/off event alone is insufficient to determine the motion direction.

To estimate local translatory motion we assume throughout the paper that the gray level function remains constant within a small neighborhood in space and time, i.e., *g*(*x*, *y*; *t*) = *g*(*x* + Δ*x*, *y* + Δ*y*; *t* + Δ*t*) (gray level constancy; c.f. Horn and Schunck, 1981). Note that due to the low latency of 15 μs of the event-based sensor (Lichtsteiner et al., 2008), this assumption is more accurate than for conventional frame based sensors. Local expansion up to the second order yields the constraint Δ*x*^*T*^ ∇_3*g*_ + 1/2 Δ*x*^*T*^ *H*_3_ Δ*x* = 0. Here, Δ*x* = (Δ*x*, Δ*y*, Δ*t*)^*T*^, ∇_3*g*_ = (*g*_*x*_, *g*_*y*_; *g*_*t*_)^*T*^ is the gradient with the 1st order partial derivatives of the continuous gray-level function, and *H*_3_ denotes the Hessian with the 2nd order partial derivatives of the continuous gray-level function that is defined in the *x*–*y*–*t*-domain. If we further assume that the 2nd order derivative terms are negligible (linear terms dominate) we arrive at the spatio-temporal constraint equation that has been used for least-squares motion estimation. The least-squares formulation is based on a set of local constraint measures over a small neighborhood under the assumption of locally constant translations (Lucas and Kanade, 1981), i.e., *g*_*x*_ *u* + *g*_*y*_ *v* + *g*_*t*_ = 0 given that Δ*t* → 0 and *u*^*T*^ = (*u*, *v*) = (Δ*x*/Δ*t*, Δ*y*/Δ*t*). Note that this motion constraint equation can also be represented in the frequency domain in which *f*_*x*_ *u* + *f*_*y*_ *v* + *f*_*t*_ = 0 holds with *f* denoting the frequency with subindices referring to the respective cardinal axes and assuming a non-vanishing energy spectrum for the gray-level luminance signal, i.e., ‖$\hat{G}$‖ ≠ 0. The local image motion *u* of an extended contrast can only be measured orthogonal to the contrast (normal flow, Wallach, 1935; Barron et al., 1994; Fermüller and Aloimonos, 1995; Wuerger et al., 1996). For simplicity, we assume a vertically oriented gray level edge (*g*_*y*_ = 0). Then the motion can be estimated along the horizontal directions (left or right with respect to the tangent orientation of the contrast edge). When the edge contrast polarity is known (light-dark, LD, *g*_*x*_ < 0 or dark-light, DL, *g*_*x*_ > 0) the spatio-temporal movements can be estimated without ambiguity. For an DL edge if *g*_*t*_ < 0 the edge moves to the right, while for *g*_*t*_ > 0 the edge moves to the left (c.f. Figure 2).

For an LD edge the sign of the temporal derivatives *g*_*t*_ changes for both respective movement directions, i.e., only the ratio of gray-level derivatives yields a unique direction selector orthogonal to the oriented luminance contrast. This means that, sgn(*g*_*x*_/*g*_*t*_) = −1 implies rightward motion while sgn(*g*_*x*_/*g*_*t*_) = 1 implies leftward motion, irrespective of the contrast polarity. Note, however, that an estimate of *g*_*x*_ is not easily accessible from the stream of events of an asynchronous event sensor. Thus, a key question is to what extend the required spatio-temporal derivative information is available and can be estimated.

#### 2.1.2. Moving gray-level edges and the spatio-temporal contrast model

We describe the luminance function *g* for a stationary DL transition by convolving a step edge 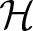
(·) with a parameterized Gaussian, 
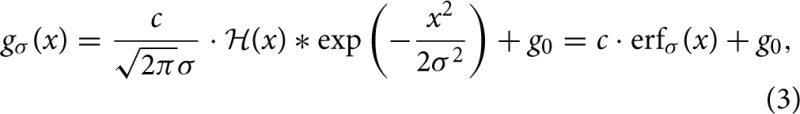
 with *c* denoting the luminance step height, *g*_0_ the basic luminance level, and “*” denoting the convolution operator (since we only study the derivatives, we assume *g*_0_ = 0). The parameter σ controls the spatial blur of the luminance edge with σ → 0 resulting in the step-function. Different contrast polarities are defined by *g*^*DL*^_σ_(*x*) = *c* · erf_σ_(*x*) and *g*^*LD*^_σ_(*x*) = *c* · (1 − erf_σ_(*x*)), respectively (Neumann and Ottenberg, 1992).

**Figure 3:**
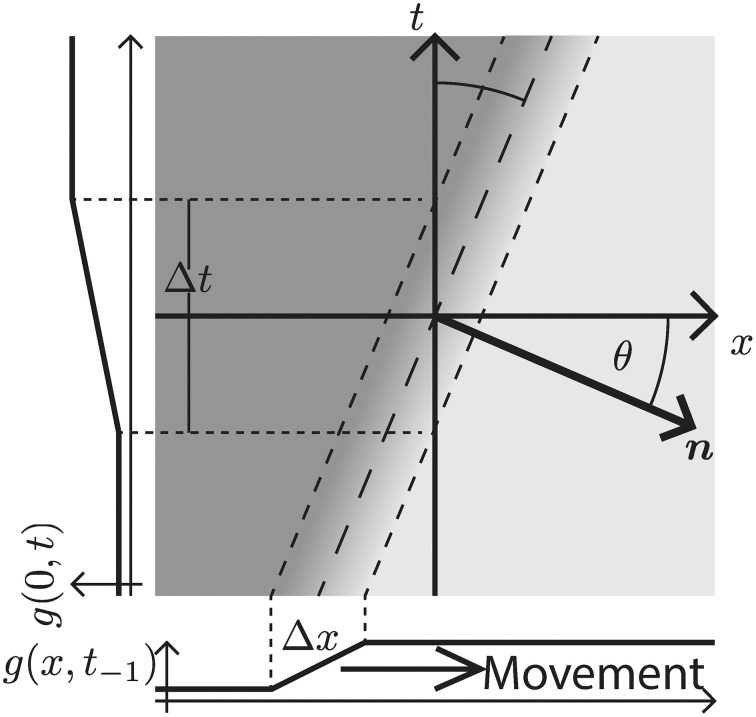
**Rightward moving 1D edge illustrated in the *x*–*t*-domain**. The velocity is defined by the direction and the speed of the spatio-temporal change. In the case depicted here, the direction is to the right and the speed is encoded by the angle θ between the *x*-axis and the normal vector *n* along the spatio-temporal gradient direction (measured in counter-clockwise rotation). Alternatively, for a contrast edge of known finite transition width Δ*x*, the speed can be inferred from the time Δ*t*, it takes the contrast edge to pass a specific location on the *x*–axis.

When this gray-level transition moves through the origin at time *t* = 0 it generates a slanted line with normal *n* in the *x*–*t*-space (c.f. Figure 3). The speed *s* of the moving contrast edge is given by *s* = sin(θ)/cos(θ), where θ is the angle between *n* and the *x*-axis (this is identical to the angle between the edge tangent and the *t*–axis). For a stationary gray-level edge (zero speed) we get θ = 0 (i.e., the edge generated by the DL transition in the *x*–*t*-domain is located on the *t*-axis). Positive angles θ ∈ (0°, 90°) (measured in counterclockwise direction) define leftward motion, while negative angles define rightward motion. For illustrative purposes, we consider an DL contrast that is moving to the right (c.f. Figure 3). The spatio-temporal gradient is maximal along the normal direction *n* = (cos θ, sin θ)^*T*^. The function *g*(*x*; *t*) describing the resulting space-time picture of the movement in the *x*-*t*-space is thus given as 
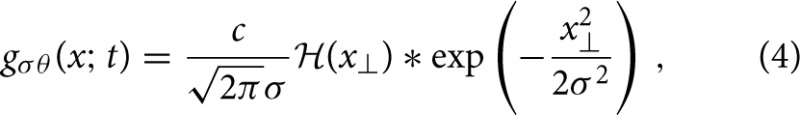
 with *x*_⊥_ = *x* · cos θ − *t* · sin θ. The respective partial temporal and spatial derivatives are given as

(5) $$\frac{\partial }{\partial t}g_{\sigma \theta }(x;t)=\frac{-c}{\sqrt{2\pi }\sigma }\exp\left(-\frac{x_{⊥}^{2}}{2\sigma^{2}}\right)\;\cdot \;\sin\theta ,$$

(6) $$\frac{\partial }{\partial x}g_{\sigma \theta }(x;t)\;=\frac{c}{\sqrt{2\pi }\sigma }\exp\left(-\frac{x_{⊥}^{2}}{2\sigma^{2}}\right)\;\cdot \;\cos\theta .$$

Now, recall that the event-based DVS sensor provides an estimate of *g*_*t*_ at a specific location [c.f. Equation (2)]. For a moving contrast profile this leads to a changing luminance function along the *t*-axis (side graph *g*(0, *t*) in Figure 3). The temporal derivative of this profile is formally denoted in Equation (5). Given a *known velocity* specified by θ, we can combine equations (5) and (6) to determine *g*_*x*_ as

(7) $$\frac{\partial }{\partial x}g_{\sigma \theta }(x;t)=-\frac{\partial }{\partial t}g_{\sigma \theta }(x;t)\;\cdot \;\tan\theta .$$

In sum, the temporal edge transition can be reconstructed in principle from a (uniform) event sequence at the edge location for a specific motion direction, given that

- a reliable speed estimate is available to infer a robust value for θ, and
- reliable estimates of temporal changes have been generated as an event cloud over an appropriately scaled temporal integration window Δ*w*_*t*_.

Note, that both parameters, θ and Δ*w*_*t*_, need to be precisely estimated to accomplish robust estimates of contrast information of the luminance edge. In Sections 2.1.4 and 2.1.5, we will briefly outline the necessary steps in such an estimation process. Alternatively, one can try to directly estimate the partial derivatives used in the motion constraint equation from the stream of events. The construction of this approach and its related problems are described in the following Section 2.1.3.

#### 2.1.3. Estimating spatio-temporal continuity using event-sequences

The local spatio-temporal movement of a gray-level function can be estimated by least-squares optimization from a set of local contrast measurements which define intersecting motion constraint lines in velocity space (Lucas and Kanade, 1981). Given a dense temporal sampling the spatio-temporal gray-level function can be reasonably well captured by a first-order approximation (as summarized in Section 2.1.1). The key question remains how one could estimate the spatial and temporal derivatives in the constraint equations, *g*_*x*_ *u* + *g*_*y*_ *v* + *g*_*t*_ = 0 from event sequences generated by the DVS. Events only encode information about the temporal derivative *g*_*t*_ [c.f. Equation (2)]. Thus, without additional information it is impossible to reliably estimate *g*_*x*_ or *g*_*y*_, as outlined in the previous Section 2.1.2. The derivative of a translatory moving gray level patch, however, generates a unique response in *h*: = *g*_*t*_. Thus, we can apply the motion constraint equation to the function *h* and solve *h*_*x*_ *u* + *h*_*y*_ *v* + *h*_*t*_ = 0, instead. Using two temporal windows 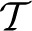
_−2_ = (*t* − 2Δ*t*, *t* − Δ*t*] and 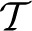
_−1_ = (*t* − Δ*t*, *t*], we can approximate *h*_*t*_, for example, by a backward *temporal* difference 
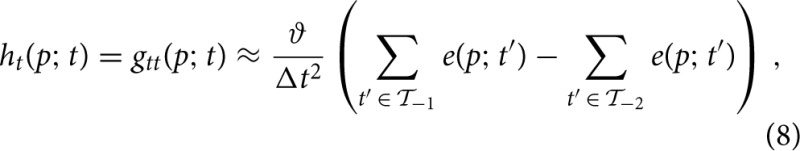
 with *p* = (*x*, *y*)^*T*^ and ϑ denoting the event-generation threshold. The *spatial* derivatives *h*_*x*_ and *h*_*y*_ can be approximated by central difference kernels [−1, 0, 1] and [−1, 0, 1]^*T*^, respectively. These can be applied to the function *h* estimated by integrating over the temporal window 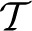 (e.g., 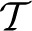 = 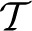
_−2_ ∪ 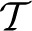
_−1_) 
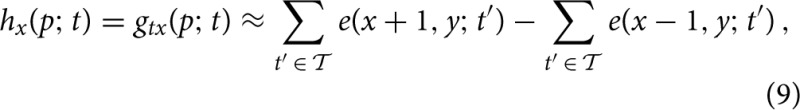
 
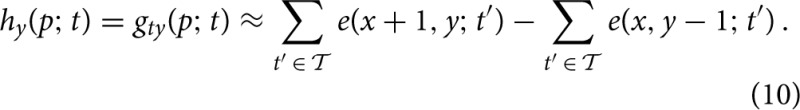


Consequently, the resulting flow computation results in a sparsification of responses since stationary edges will not be represented in *h*. This approach is similar to that of Benosman et al. (2012) but consistently employs the second derivative instead of mixing the first and second derivatives which leads to inconsistencies in general.

Note, however, that this approach has multiple issues regarding any real implementation. The most important observation is that when a luminance edge passes a pixel's receptive field of the DVS sensor, the amount of events is in the range of about 10 events (often even less, depending on the contrast, speed and luminance conditions; c.f. zoomed display of the event cloud in Figure 1). Thus, huge approximation errors occur for *h*_*x*_, *h*_*y*_ and especially in *h*_*t*_ (since this now represents the second derivative of the original gray-level function *g*). Furthermore, we can only estimate *h*_*t*_ accurately, if the temporal windows are small enough such that the gray-level edge has not already passed through the receptive field of a target cell at position *p*. This limits the number of events to even less and leads to magnifying the outlined problems even further. Alternatively, one could try to directly approximate the temporal derivative for each event by incorporating the time-span since the last event, i.e.,

(11) $$\frac{d}{dt}g(p;t)=g_{t}(p;t)\approx \frac{\vartheta }{\Delta_{W}t}e(p,t),$$

with Δ_*W*_ *t* representing the time that has passed since the last event generated at *p*. This assumes a constant intensity change since the last event. This, however, is certainly not true for the first event because first nothing happens for a long period and then occasionally some change occurs that causes the event, i.e., the estimate will be too small, because Δ_*W*_ *t* is too big.

#### 2.1.4. Least-squares velocity estimation

The short temporal window in which events of a briefly passing contrast edge are generated makes it difficult to reliably estimate the derivatives required in the motion constraint equation (c.f. previous section). An alternative approach is to consider the distribution of events (the “event cloud”) in a small volume of the *x*-*y*-*t*-space. The cloud that results from a moving contrast edge generates a locally plane-like cloud of on- and/or off-events (with on- and off-events in the case of a line, for example, and only on- or off-events in the case of a transition from one homogeneous region to another) to which a velocity tangent plane can be fitted (Benosman et al., 2014). The thickness of the event cloud orthogonal to the velocity tangent plane depends on the sharpness of the contrast edge, the speed with which the gray-level discontinuity moves through the spatial location of a pixel, and its local neighborhood (the receptive field, RF, of a cell at this position). In Benosman et al. (2014) a function Σ_*e*_ : ℕ^2^ → ℝ is defined that maps the location *p* of an event *e* to the time Σ_*e*_(*p*) = *t* when the event was generated. This mapping may be used to describe the cloud of events. However, care should be taken since the mapping is non-continous in principle: it is either defined for each event in which case the mapping is not differentiable, or it is defined for all events in which case the mapping is not injective (because for a given *t*, there are multiple events at different locations). In any case, the inverse function theorem of calculus (as employed in Benosman et al., 2014) *cannot* be applied here to derive a speed estimate. This insight might explain, why in the velocity-vector-field of a rotating bar illustrated in Figure 7b of Benosman et al. (2014) the velocity vectors at the outer parts are shorter (instead of longer) compared to the velocity vectors at the inner ones. We suggest an alternative solution in which the speed is estimated from the regression plane by solving the orthogonal system of the velocity vector *v* = (*u*, *v*, 1)^*T*^ (defined in homogeneous coordinates), the orientation of the moving luminance edge *l* = (*l*_*x*_, *l*_*y*_, 0)^*T*^, and the normal vector *n* = (*a*, *b*, *c*)^*T*^ of the plane. These three vectors form an orthogonal system that spans the *x*-*y*-*t* space:

(12) $$n⊥v\Rightarrow n^{T}\;\cdot \;v=au+bv+c\;=0$$

(13) $$n⊥l\Rightarrow n^{T}\;\cdot \;l=al_{x}+bl_{y}=0$$

(14) $$l⊥v\Rightarrow l^{T}\;\cdot \;v=ul_{x}+vl_{y}=0$$

(15) (13) · u−(14) · a⇒bu−av=0

(16) $$(12)\;\cdot \;b-(15)\;\cdot \;a\Rightarrow v\;\cdot \;(a^{2}+b^{2})+bc=0$$

(17) $$(12)\;\cdot \;a+(15)\;\cdot \;b\Rightarrow u\;\cdot \;(a^{2}+b^{2})+ac=0.$$

The resulting velocity components *u* and *v* are then given as (with *n* = (*a*, *b*, *c*)^*T*^)

(18) $$\left(\begin{array}{c} u\\ v \end{array}\right)=-\frac{c}{a^{2}+b^{2}}\left(\begin{array}{c} a\\ b \end{array}\right),$$

with the speed component $s=\sqrt{u^{2}+v^{2}}=c\;\cdot \;{\left(a^{2}+b^{2}\right)}^{-1/2}$. Note, that for slow or moderate velocities, a reliable estimate of the velocity tangent plane requires a spatial as well as a temporal neighborhood such that the event cloud is fully covered within the spatio-temporal window (or RF) considered for the LS regression. In particular, the neighborhood support must cover the event cloud illustrated in the bottom right of Figure 1. If this condition is not fulfilled, i.e., if the window is smaller than the extent of the cloud, then the principal axes are arbitrary and cannot be estimated reliably.

#### 2.1.5. Direction-sensitive filters

As an alternative to considering the LS regression in estimating the velocity tangent plane from the cloud of events, the uncertainty of the event detection might be incorporated directly. At each location, detected events define likelihood distributions *p*(*e*|*u*) given certain velocities of the visual scene (estimated by a filter bank, for example). Using Bayes' theorem, we derive that for each event *p*(*u*|*e*) ∝ *p*(*e*|*u*) · *p*(*u*). If each velocity is equally likely to be observed without a priori knowledge, i.e., *p*(*u*_*i*_) = *p*(*u*_*j*_) (for arbitrary velocities *i*, *j*), it holds *p*(*u*|*e*) ∝ *p*(*e*|*u*) and thus, the velocity *u*_*est*_ of the movement that caused event *e* can be estimated as

(19) $$u_{est}={\text{argmax}}_{u}p(u|e)={\text{argmax}}_{u}p(e|u).$$

Thus, we can estimate the velocity from the responses *p*(*e*|*u*_*i*_), *i* = 1, 2, … of a filter bank, for example. In addition, a priori knowledge could be incorporated to reduce noise and to increase coherency. Current knowledge suggests, that such distributions are represented by the filter characteristics of the spatio-temporal receptive fields of cells in area V1 which we use as inspiration for a novel filter mechanisms described in the following Section 2.2.

### 2.2. Event-based motion estimation using direction-selective filters

In this section, we define spatio-temporal filters that are fitted to the physiological findings from De Valois et al. (2000) summarized in the following Section 2.2.1.

#### 2.2.1. Experimental evidence

Our filter design essentially reverses the decomposition of neural responses conducted by De Valois et al. (2000) (also c.f. Tschechne et al., 2014). Based on physiological findings first described by DeAngelis et al. (1995), De Valois suggested that inseparable filters stem from a combination of various separable components (De Valois et al., 2000). In De Valois et al. (2000) cortical V1 cells were tested and strong evidence for the coexistence of two distinct types of populations of cells emerged: One population showed spatio-temporally separable weight functions of either even or odd spatial symmetry. These have either temporally mono- or bi-phasic response characteristics which were mainly determined by a single principal component in 2D (of a singular value decomposition). The other population of cells was spatio-temporally inseparable showing a receptive field distribution of selectivity that were slanted with respect to the time axis, i.e., motion sensitive (c.f. Figure 3; c.f. also De Valois and Cottaris, 1998). Response characteristics of these cells were determined by *two* strong principal components in 2D. These two components of the second group were itself spatio-temporally separable with spatially out-of-phase components and always composed of pairs of mono- and bi-phasic distributions.

**Figure 4:**
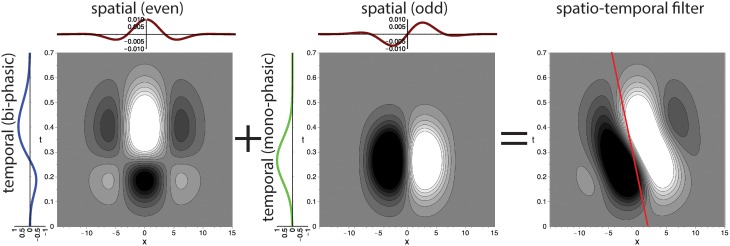
**Superposition of spatio-temporally separable filters creates motion-direction sensitive filter**. The proposed spatio-temporal filter (right) is constructed by using the results of the singular value decomposition of the receptive field of motion directional cells by De Valois et al. (2000) (c.f. DeAngelis et al., 1995, their Figure 3). Two separable filters are superposed to create the final motion direction selective spatio-temporal filter. Each of the two filters is separable into a pair of a bi-phasic temporal and an even spatial or a mono-phasic temporal and an odd spatial filter, respectively (illustrated by line profile plots to the left and at the top). The red line indicates the preferred speed selectivity identified by a Fourier analysis of the filter function (c.f. Section 2.2.4).

This main observation lead us to propose a family of spatio-temporally direction selective filters as illustrated in Figure 4, that are generated by superposed separable filters with quadrature pairs of spatial weighting profiles (*G*_*odd*_ and *G*_*even*_) and mono-/bi-phasic temporal profiles (*T*_*mono*_ and *T*_*bi*_). The details of the construction process are outlined in the following sections.

#### 2.2.2. Spatial gabor filters

**Figure 5:**
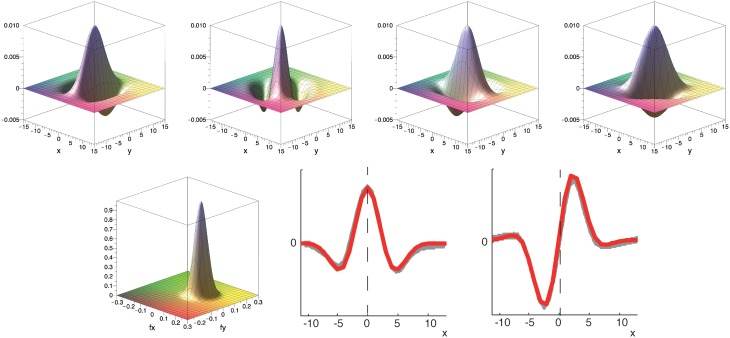
**Top row:** Example of filter bank of four oriented Gabor filters for Φ = [0°, 45°, 90°, 135°] (to resemble the spatial receptive field weights; only even component is shown). **Bottom row:** Corresponding filter in Fourier domain and illustration of spatial fitting of analytical (red) and experimental data (gray) from De Valois et al. (2000). Parameters in all plots have been set to σ = 25 and *f*_0_ = 0.08.

To construct the spatial component of the spatio-temporal filters illustrated in Figure 4 we define Gabor filters that are fitted to the experimental results of De Valois et al. (2000). To construct multiple spatio-temporally tuned filters of different spatial orientation selectivity, we employ a filter-bank of kernels as illustrated in Figure 5. More precisely, we employ Gabor filters maximally selective for the spatial frequency (*f*^0^_*x*_, *f*^0^_*y*_) (with a standard deviation σ in local space) defined by (c.f. Figure 5)

(20) $$\begin{array}{l} G_{\sigma ,f_{x}^{0},f_{y}^{0}}(x,y)=\frac{2\pi }{\sigma^{2}}\;\cdot \;\exp\left[2\pi j\left(f_{x}^{0}x+f_{y}^{0}y\right)\right]\cdot \\ \;\exp\left[-\frac{2\pi^{2}\;\cdot \;\left(x^{2}+y^{2}\right)}{\sigma^{2}}\right], \end{array}$$

in local space. The spatial frequencies selected by this filter can be seen by visualizing its Fourier transform (Figure 5, bottom left) which is given as

(21) $${\hat{G}}_{\;\hat{\sigma },f_{x}^{0},f_{y}^{0}}(f_{x},f_{y})=\exp\left[-\frac{1}{2}\;\cdot \;\frac{{\left(f_{x}-f_{x}^{0}\right)}^{2}+{\left(f_{y}-f_{y}^{0}\right)}^{2}}{{\hat{\sigma }}^{2}}\right],$$

where $\hat{\sigma }$ = 1/σ and the filter tuning (*f*^0^_*x*_, *f*^0^_*y*_) defines the shift of the Gaussian envelope with respect to the origin in the Fourier domain. This defines the two components *G*_*odd*_ = ℑ(*G*_σ, *f*^0^_*x*_, *f*^0^_*y*__) and *G*_*even*_ = ℜ(*G*_σ, *f*^0^_*x*_, *f*^0^_*y*__) to construct the filters as described in Section 2.2.1 (compare with Daugman, 1985; Marčelja, 1980).

#### 2.2.3. Mono- and biphasic temporal filters

**Figure 6:**
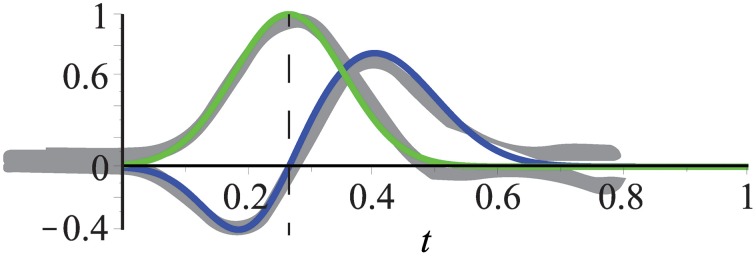
**Temporal filters fit experimental data (gray) from De Valois et al. (2000)**. Kernels consist of one or two Gaussians which define a mono- and bi-phasic temporal filter, respectively. The mean of the mono-phasic kernel has been set to μ_*bi*1_ = 0.2 with the remaining parameters fitted to the experimental data (see text for details). Dashed line highlights that the peak of the mono-phasic kernel (green) is located at the zero-crossing of the bi-phasic kernel (blue).

The second component required in the spatio-temporal filter generation process illustrated in Figure 4 is the definition of mono- and bi-phasic temporal filters, *T*_*mono*_ and *T*_*bi*_. To fit the experimental data of De Valois et al. (2000), we define (c.f. Figure 6)

(22) $$T_{mono}(t)=G_{\sigma_{mono},\mu_{mono}}(t),$$

(23) $$T_{bi}(t)=-s_{1}\;\cdot \;G_{\sigma_{bi1},\mu_{bi1}}(t)+s_{2}\;\cdot \;G_{\sigma_{bi2},\mu_{bi2}}(t),$$

with the unnormalized Gaussian function

(24) $$G_{\sigma ,\mu }(t)=\exp\left(-\frac{{\left(t-\mu \right)}^{2}}{2\sigma^{2}}\right).$$

When the experimental findings are incorporated, it is only necessary to choose a value for μ_*bi*1_. All other parameters can be inferred according to the experimental data from De Valois et al. (2000):

- The bi-phasic scaling factors *s*_1_ and *s*_2_ are adapted to the minimum and maximum values of the experimental data relative to the maximum value of the monophasic kernel (which is one), i.e., *s*_1_ = 1/2 and *s*_2_ = 3/4.
- A good fit with the experimental data reported in De Valois et al. (2000) is achieved by setting the relation between the mean values to μ_*bi*2_ = 2μ_*bi*1_.
- The standard deviations σ_*mono*_ and σ_*bi*1_ are chosen such that the Gaussians are almost zero for *t* = 0, i.e., σ_*mono*_ = μ_*mono*_/3, σ_*bi*1_ = μ_*bi*1_/3 (3σ–rule; 99.7% of the values lie within three standard deviations of the mean in a normal distribution).
- The standard deviation of the second Gaussian of the bi-phasic kernel is about 3/2 of that of the first, i.e., $\sigma_{bi2}=\frac{3}{2}\;\cdot \;\sigma_{bi1}=\frac{1}{2}\;\cdot \;\mu_{bi1}$.
- The mean of the mono-phasic kernel μ_*mono*_ is given by the zero-crossing of the biphasic kernel, i.e., $\mu_{mono}=\frac{1}{5}\;\cdot \;\left(1+\mu_{bi1}\;\cdot \;\sqrt{36+10\;\cdot \;\ln\left(s_{1}/s_{2}\right)}\right)$.

Figure 6 illustrates that these settings result in a good fit of the temporal filters with the experimental data reported in De Valois et al. (2000). We will now construct the full spatio-temporal selective filters as outlined in Figure 4.

#### 2.2.4. Combined spatio-temporal filter

**Figure 7:**
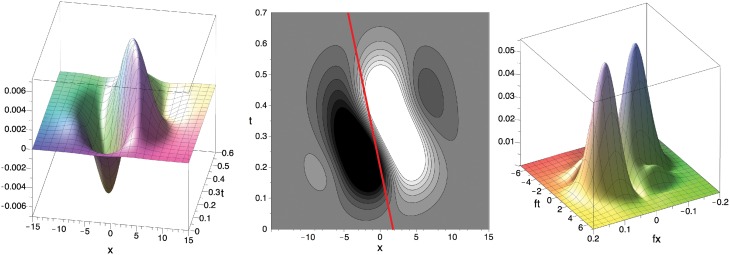
**Filter *F* in *x*–*t*–space (left, center; c.f. DeAngelis et al. (1995), Their Figure 3F) and in Fourier domain (right; absolute value) for σ = 25, *f*^0^_*x*_ = *f*^0^_*y*_ ≈ 0.057 ($f_{0}=\sqrt{{\left(f_{x}^{0}\right)}^{2}+{\left(f_{y}^{0}\right)}^{2}}=0.08$) and μ_*bi*1_ = 0.2 (as in Figure 4)**. Red line illustrates the velocity component in the *x*–*t*-domain (≈ 8.61 *pixel*/*s*) inferred from the values maximizing the absolute value of the Fourier transform |$\hat{F}$| of the filter *F* (*f*^*max*^_*t*_ ≈ 0.965, *f*^*max*^_*x*_ ≈ *f*^*max*^_*y*_ ≈ 0.057). See text for details.

The full spatio-temporal filter *F* is defined according to the scheme of Figure 4, i.e., by the sum of two products consisting of the odd-spatial *G*_*odd*_ = ℑ(*G*_σ, *f*^0^_*x*_, *f*^0^_*y*__), the monophasic temporal *T*_*mono*_, the even-spatial *G*_*even*_ = ℜ(*G*_σ, *f*^0^_*x*_, *f*^0^_*y*__), and the biphasic temporal filter *T*_*bi*_ (c.f. Figure 7):

(25) $$F(x,y,t)=ℑ(G_{\sigma ,f_{x}^{0},f_{y}^{0}}(x,y))\;\cdot \;T_{mono}(t)+ℜ(G_{\sigma ,f_{x}^{0},f_{y}^{0}}(x,y))\;\cdot \;T_{bi}(t).$$

The preferred speed of the filter can be determined by an analysis of the Fourier transform $\hat{F}$(*f*_*x*_, *f*_*y*_, *f*_*t*_) of the filter function *F*(*x*, *y*, *t*). From the location (*f*^*max*^_*t*_, *f*^*max*^_*x*_, *f*^*max*^_*y*_) where $\hat{F}$ is maximal we can infer the filter's preferred normal velocity, i.e., the velocity parallel to the gradient of the luminance edge (*n* in Figure 3) with maximal filter response, using the following two relations:

- The motion constraint equation in the frequency domain: *f*_*x*_ *u* + *f*_*y*_ *v* + *f*_*t*_ = 0, i.e., *f* · *u* = −*f*_*t*_.
- (*u*_⊥_,*V*_⊥_) is orthogonal to the luminance edge, i.e., parallel to (*f*^*max*^_*x*_, *f*^*max*^_*y*_). Thus, the scalar product of *f*^*max*^ = (*f*^*max*^_*x*_, *f*^*max*^_*y*_) and *u*_⊥_ = (*u*_⊥_,*V*_⊥_) is equal to *f*^*max*^ · *u*_⊥_ = ‖*f*‖· ‖*u*‖.

Combining both equations, we obtain −*f*^*max*^_*t*_ = ‖*f*^*max*^‖ · ‖*u*_⊥_‖, i.e., the speed *s* = ‖*u*_⊥_‖ is given as *s* = ‖*u*_⊥_‖ = −*f*^*max*^_*t*_/‖*f*^*max*^‖. The velocity can now be obtained by scaling the normalized gradient direction *f*^*max*^/‖*f*^*max*^‖ with $\|u_{⊥}\|=\sqrt{u_{⊥}^{2}+v_{⊥}^{2}}$ gaining

(26) $$u_{⊥}=-\frac{f_{t}}{{\left(f_{x}^{max}\right)}^{2}+{\left(f_{y}^{max}\right)}^{2}}\;\cdot \;f_{x},$$

(27) $$v_{⊥}=-\frac{f_{t}}{{\left(f_{x}^{max}\right)}^{2}+{\left(f_{y}^{max}\right)}^{2}}\;\cdot \;f_{y},$$

in *pixel*/*s*. For the parameter values that fit the experimental data from (De Valois et al., 2000), i.e., σ = 25, *f*^0^_*x*_ = *f*^0^_*y*_ ≈ 0.057 ($f_{0}=\sqrt{{\left(f_{x}^{0}\right)}^{2}+{\left(f_{y}^{0}\right)}^{2}}=0.08$) and μ_*bi*1_ = 0.2, we numerically determined the values as *f*^*max*^_*t*_ = 0.974, *f*^*max*^_*x*_ = *f*^*max*^_*y*_ = 0.057 which maximize |$\hat{F}$|. Thus, the fitted spatio-temporal selective filter *F* is maximally selective for the velocity (*u*_⊥_,*V*_⊥_) = (8.61, 8.61)*pixel*/*s*, i.e., a speed of 12.2 *pixel*/*s*.

#### 2.2.5. Response normalization

The spatio-temporal filter mechanism is combined with a stage of down-modulating lateral divisive inhibition. Such response normalization was shown to have a multitude of favorable properties such as the decrease in response gain and latency observed at high contrasts, the effects of masking by stimuli that fail to elicit responses of the target cell when presented alone, the capability to process a high dynamic range of response activations (Heeger, 1992; Carandini et al., 1997; Koch, 1999; Sceniak et al., 1999; Frégnac et al., 2003; Tsui et al., 2010), and the ability to resolve ambiguous motion estimates at, for example, straight contours without knowledge about the edges of the contour (aperture problem Wallach, 1935; Nakayama and Silverman, 1988; Wuerger et al., 1996). To account for such nonlinearities we add a stage of divisive normalization to test whether it is also suited to enhance flow estimated from the output of DVSs. Based on our previous modeling (e.g., Raudies et al., 2011; Brosch and Neumann, 2012, 2014a), we employ a dynamic neuron model of membrane potentials *p* and a mean firing rate generated by the monotonically increasing function Ψ(*p*). The full dynamic equation reads

(28) $${\dot{p}}_{i}=-\alpha_{p}p_{i}+(\beta -p_{i})\;\cdot \;I_{i}-p_{i}\;\cdot \;\Psi_{q}(q_{i}),$$

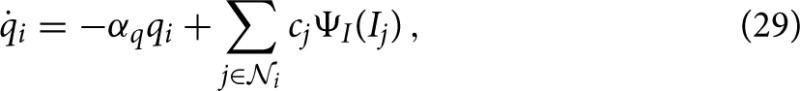
 with *I*_*i*_ denoting the input and *c*_*j*_ denote the spatio-temporal weighting coefficients of the local neighborhood 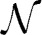
_*i*_ of neuron *i* in the space-time-feature domain (see Brosch and Neumann, 2014a for more details of an even more generalized circuit model). At equilibrium, the following state equations can be derived 
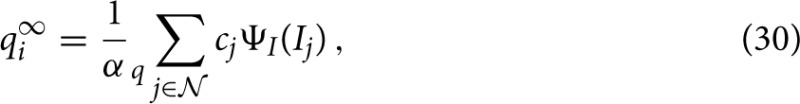
 
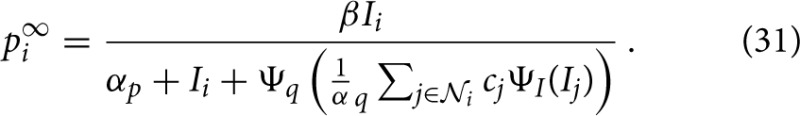


Another favorable property of divisive normalization has been the observation that it can approximate a process dubbed *radial Gaussianization* which minimizes the statistical dependency of coefficients in image coding (Lyu and Simoncelli, 2008b, 2009a):

(32) $${\left(p_{norm}\right)}_{i}\frac{I_{i}}{{\left(b+\sum_{j}c_{j}I_{j}^{2}\right)}^{1/2}},$$

where *b* is a scalar scaling coefficient and *c*_*j*_ denote the weighting coefficients for the activations in the surrounding neighborhood in the space-feature domain [as in Equation (29)]. When the coefficients are learned from a test set (Lyu and Simoncelli, 2009a), it was shown to approximate optimal minimization of statistical dependency, i.e., radial Gaussianization. Here, we test whether this is also true for Gaussian weights (in accordance with neurophyiological findings Bonin et al., 2005) and a slightly different but biologically inspired normalization scheme as outlined in Equation (31). Therefore, the normalization scheme adopted here can only lead to an approximate decorrelation of input encoding. We will, therefore, demonstrate experimentally the impact of the divisive normalization of the spatio-temporal input filtering.

## 3. Results

In addition to the main part describing the theoretical investigations outlined in the previous sections, we conducted a series of experiments to validate the modeling approach and its theoretical properties. The parameters of the spatio-temporal filters were chosen such that they fit the experimental data as reported in De Valois et al. (2000) (up to scaling), namely μ_*bi*1_ = 0.2 for the temporal filter components, and σ = 25, *f*_0_ = 0.08 for the spatial filter components. The parameters of the normalization mechanism in Equation (31) were set to β = 1, α_*p*_ = 0.1, α_*q*_ = 0.002, *c*_*j*_ resemble the coefficients of a Gaussian kernel with σ = 3.6, and Ψ_*I*_(*x*) = Ψ_*q*_(*x*) = max(0, *x*) denotes a rectifying transfer function.

First, we probed the model using simple and more complex stimuli with translatory and rotational motion to demonstrate the detection performance and noise characteristics of the initial (linear and non-linear filtering of the input). Second, we studied the impact of the normalization stage on the initial filter responses. Third, the model was probed by stimuli with transparent overlaid motion patterns to test the segregation into multiple motion directions at a single spatial location (see e.g., Braddick et al., 2002; Edwards and Nishida, 1999; Treue et al., 2000).

### 3.1. Detection of translatory and rotational movements

**Figure 8:**
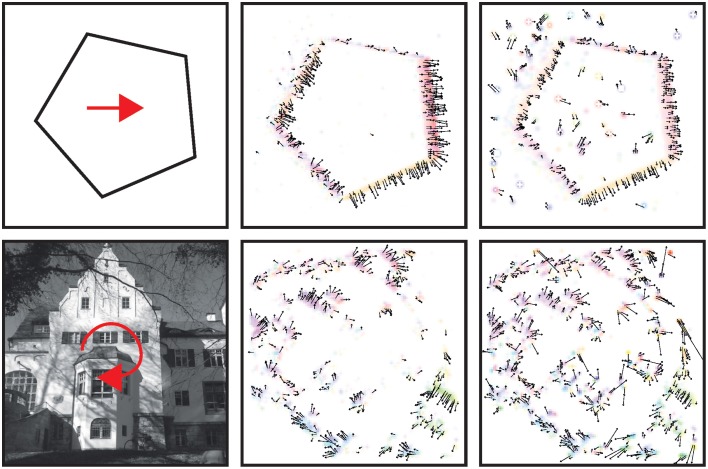
**Responses to input stimuli with translatory and rotational motion**. From left to right: Test stimulus and vector field of initial motion estimation using the filter mechanism in Equation (25) and after normalization (red arrows are not part of stimulus; only two representative stimuli are shown due to space constraints). **First row:** Translatory motion stimulus illustrates that a majority of the responses point into the normal flow-direction, i.e., orthogonal to the stimulus boundaries. **Last row:** A rotational stimulus has been employed to validate that the filter also works for different speeds (slow motion close to the center and fast motion at the more distant regions). See Section 3.2 for details about the normalization mechanism. A comparison of initial and normalized flow estimation demonstrates that responses within line segments are reduced while responses at corners or noise are enhanced (that could be compensated by feedback from higher stages Brosch and Neumann, 2014b).

At each location the filter creates a population code of length *N* with each entry corresponding to the response of a spatio-temporal filter with motion direction selectivity θ_*k*_. For visualization purposes (Figure 8), the velocity components *u*_*p*_ and *v*_*p*_ are inferred from the initial responses *I*_*p*; *k*_, *k* ∈ {1, …, *N*} at each location *p* by summing them up according to

(33) $$\left(\begin{array}{c} u_{p}\\ v_{p} \end{array}\right)=\sum_{k\;=\;1}^{N}I_{k}\;\cdot \;\left(\begin{array}{c} \cos(2\pi (k-1)/N)\\ -\sin(2\pi (k-1)/N) \end{array}\right),$$

effectively implementing a local vector addition of component estimates. The tests utilize stimuli of translatory and rotational motion. The visualized results (Figure 8) demonstrate that the filter based approach robustly computes estimates of contour motion, i.e., locations of apparently moving contrasts and object boundaries (Barranco et al., 2014).

### 3.2. Response normalization

**Figure 9:**
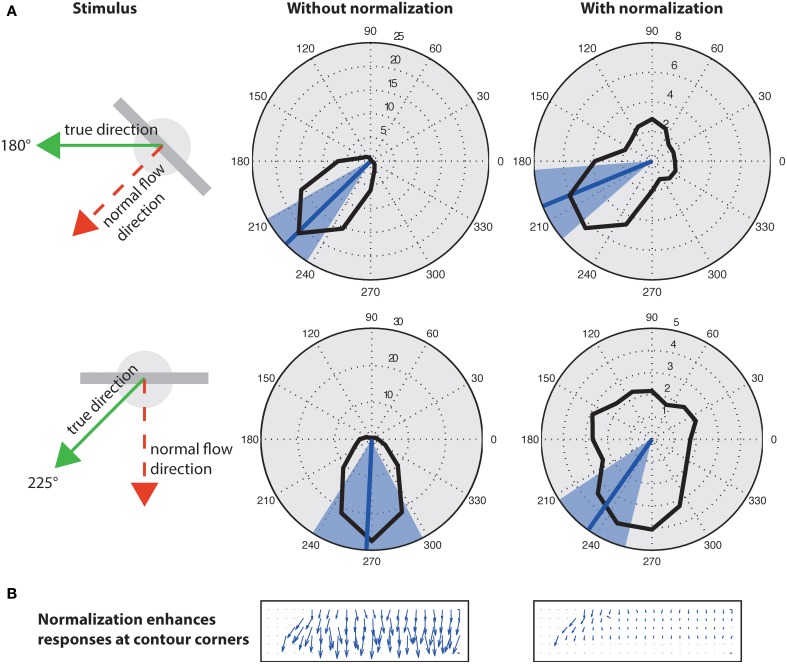
**Effect of normalization on initial flow estimation of two oblique bars moved with 45° difference to their orientation**. **(A)**, Left: Initial estimates elicit maximum response for normal flow direction (blue line; blue areas indicates standard deviation). Right: Surround inhibition enhances responses at the corners, effectively biasing the estimate toward the true motion direction. The circles on the elongated bar stimulus show the size of kernel weighting functions. **(B)** Normalization suppresses responses “within” the line and enhances responses at its endings (line ends, corners) that encode the true motion direction (c.f. Guo et al., 2006; Tsui et al., 2010).

A well known problem to motion detection is the estimation of ambiguous motion at e.g., straight contours (aperture problem). Locally only the normal flow direction can be measured which might not coincide with the true direction because the motion component parallel to a contrast edge is unknown (Figure 9, left). As suggested in Tsui et al. (2010), normalization can help to suppress responses at ambiguous parts of a contour (i.e., the inner parts of an extended contrast or line) and to enhance responses at line ends or sharp corners (c.f. Figure 9B). Figure 9 shows motion histograms of the tilted bar in Figure 8 (top) as a result of the initial filtering in the model (left) and with normalization (right). These results indicate that normalization significantly improves the histograms to better represent the true motion direction (Figure 9A; blue lines).

In Section 2.2.5, we point out that divisive normalization can effectively approximate radial Gaussianization, i.e., a reduction of the dependency between components within a population code. Here, we empirically validate that the divisive normalization described in Equation (31) indeed reduces the dependency within the population of motion selective cells. We quantify the statistical dependency of the multivariate representation by using multi-information (MI) (Studený and Vejnarová, 1998), which is defined as the Kullback-Leibler divergence (Cover and Thomas, 2006; Lyu and Simoncelli, 2009a) between the joint distribution *p*(*x*_1_, *x*_2_, …, *x*_*d*_) and the product of its marginals 
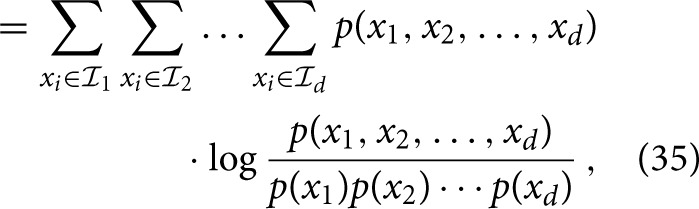

(35) $$MI(I)=D_{KL}\left(p(I)\|\prod_{k}p(I_{k})\right)=\left[\sum_{k\;=\;1}^{d}H(I_{k})\right]-H(I)$$

where *H*(*I*) = ∫ *p*(*I*)log(*p*(*I*)) *dI* is the differential entropy of the representation *I*, and *H*(*I*_*k*_) denotes the differential entropy of the *k*th component of *I* (Lyu and Simoncelli, 2009a). To calculate the required probability estimates, we employ binary variables indicating motion for *d* = 4 movement directions. As theoretically predicted by the connection to radial Gaussianization, the MI for the stimulus shown in Figure 9 is reduced from *MI*(*I*) = 0.042 (0.090 for the second example) before normalization to *MI*(*I*_*norm*_) = 0.028 (0.027 for the second example) after the normalization stage. Thus, divisive normalization employed here does not entirely decorrelate the movement representation (which would imply *MI*(*I*_*norm*_) = 0) but significantly reduces it.

### 3.3. Spatio-temporal filtering and transparent motion

**Figure 10:**
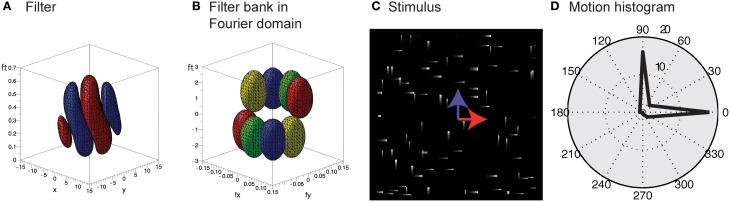
**Encoding of motion transparency in the proposed model**. **(A)** Illustration of a single spatio-temporal filter (surfaces indicate that *F* = ± 0.0005 for red/blue). Note that this filter resembles measurements of, for example, the cat's striate cortex (e.g., DeAngelis et al., 1995, Their Figure 2B). **(B)** Illustration of the preferred frequencies (surfaces indicate that |$\hat{F}$| = 0.1) of four filters of a filter bank in the Fourier domain [red pair of ellipsoids corresponds to Fourier spectrum of the filter shown in **(A)**]. Note that the combined ellipsoids sample the frequency space, with each pair responding to a certain speed and motion direction. **(C)** Stimulus consisting of a random dot pattern of dots moving to the right or to the top with equal speeds. **(D)** Motion histogram of filter responses. While it is not possible to fit a plane to the resulting event cloud, the proposed filter based approach encodes both movement directions.

Unlike the motion of opaque surfaces transparent motion is perceived when multiple motions are presented in the same part of visual space. Few computational model mechanisms have been proposed in the literature that allow to segregate multiple motions (see e.g., Raudies and Neumann, 2010; Raudies et al., 2011 which include recent overviews). All such model approaches are based on frame-based inputs. For that reason, we investigate how transparent motion induced by random dot patterns moving in different directions is represented in event-clouds originating from DVSs. In general, filter-based mechanisms are able to encode estimated motions for multiple directions at a single location. In contrast, it is not possible to fit a plane at positions where two (or multiple) event clouds generated by, for example, two crossing pedestrians intersect without applying additional knowledge. The filter mechanisms proposed in this work naturally encode motion directions within the uncertainty of the integration fields (c.f. Figures 10A,B). In order to build such a filter bank, the frequency space in Figure 10B needs to be sampled properly in accordance with the theoretical analysis outlined in Section 2 (c.f. Table 1).

**Table 1:** **Effect of different settings of parameters *f*_0_ and μ_*bi*_ on the speed selectivity (in *pixel*/*s*), i.e., *f*^0^_*t*_/*f*^0^_*x*_, with *f*^0^_*x*_ and *f*^0^_*t*_ maximizing |$\hat{F}$| for σ = 25**.

***f*_0_\μ_*bi*_**	**0.05**	**0.10**	**0.15**	**0.20**	**0.25**	**0.30**	**0.35**	**0.40**	**0.45**
0.04	100.65	50.33	33.55	25.16	20.13	16.78	14.38	12.58	11.18
0.05	78.76	39.38	26.26	19.69	15.75	13.13	11.25	9.85	8.75
0.06	65.10	32.55	21.70	16.28	13.02	10.85	9.30	8.14	7.23
0.07	55.67	27.84	18.56	13.92	11.13	9.28	7.95	6.96	6.18
0.08	48.69	24.34	16.23	12.17	9.74	8.11	6.96	6.09	5.41
0.09	43.28	21.64	14.42	10.82	8.66	7.21	6.18	5.41	4.81
0.10	38.95	19.48	12.98	9.74	7.79	6.49	5.56	4.87	4.33
0.11	35.41	17.70	11.80	8.85	7.08	5.90	5.06	4.43	3.93
0.12	32.46	16.23	10.82	8.11	6.49	5.41	4.64	4.06	3.61

The different parameter configurations allow to realize filter banks selective for a wide range of different speeds. If, for example, motion with a speed of about 10 pixels/s needs to be detected, the table reveals that the parameters can be chosen as (*f*_0_, μ_*bi*_) = (0.10, 0.20) or as (0.05, 0.40), for example. Further adaptation of the standard deviations of the spatial and temporal kernels according to our theoretical results allows to realize optimal sampling of the Fourier-domain. For large enough σ (as for this table), the speed-selectivity does hardly depend on the parameter σ. For small σ, however, we noticed a strong impact which needs to be considered in the creation of a properly tuned filter bank.

To test the encoding of motion transparency, we probed the model by using simulated event-based sensor outputs of two superimposed random-dot patterns moving in orthogonal directions with the same speed. The spatio-temporal event-cloud generated by the moving dots is rather noisy and the component motions appear rather indistinguishable by eye. Figure 10C shows such events for individual dots and integrated over a small temporal window (directions are indicated by the blue and red arrows for illustrative purposes). As can be seen in Figure 10D the filter response clearly encodes both movement directions which could not be achieved by a plane-fitting approach without incorporating knowledge about the number of movement directions.

## 4. Discussion

This paper investigates mechanisms for motion estimation given event-based input generation and representation. The proposed mechanism has been motivated from the perspective of sampling the plenoptic function such that specific temporal changes in the optic array are registered by the sensory device. The temporal sampling is based on significant changes in the (log) luminance distribution at individual sensory elements (pixels). These operate at a very low latency by generating events whenever the local luminance function has undergone a super-threshold increment or decrement. This is fundamentally different from common frame-based approaches of image acquisition where a full image is recorded at fixed intervals leading to a largely redundant signal representations. Our focus is on motion computation and the proposed approach is different from previous approaches in several respects. In a nutshell, our paper makes three main contributions:

- We first investigate fundamental aspects of the local structure of lightfields for stationary observers and local contrast motion of the spatio-temporal luminance function. In particular, we emphasize the structure of local contrast information in the space-time domain and their encoding by events to build up an address-event representation (AER).
- Based on these results we derive several constraints on the kind of information that can be extracted from event-based sensory acquisition using the AER principle. This allows us to challenge several previous approaches and to develop a unified formulation in a common framework of event-based motion detection.
- We have shown that response normalization as part of a canonical microcircuit for motion detection is also applicable for event-based flow for which it reduces motion ambiguity and contributes to making the localized measures of filtering statistically more independent.

These different findings will be discussed in more detail in the following sections.

### 4.1. Previous related computational models

So far, only relatively few investigations have been published that report on how classical approaches developed in computer vision can be adapted to event-based sensory input and how the quality of the results changes depending on the new data representation framework. Examples are Benosman et al. (2012, 2014) for optical flow computation and (Rogister et al., 2012; Piatkowska et al., 2013; Camuñas Mesa et al., 2014) for stereo vision. Furthermore, other authors show future applications of this new sensor technology that have the potential to provide fast, robust and highly efficient sensory processing in various domains and challenging scenarios (e.g., Fu et al., 2008; Drazen et al., 2011). Even further, most recent work has elucidated how fast event-based sensing technology can be utilized to improve the performance of computer vision motion estimation approaches and how frame-based imagery may help stabilizing the raw event-based motion processing (Barranco et al., 2014).

We here focus on the detection of flow from spatio-temporal motion on the basis of event-based sensor input. We utilize the dynamic-vision sensor (DVS) that emulates the major processing cascade of the retina from sensors to ganglion cells (Lichtsteiner et al., 2008; Liu and Delbruck, 2010). Based on the formulation of a local spatio-temporal surface patch at a significant luminance transition that moves along either direction, we have first categorized event-based flow estimation models. This allows us to provide a more systematic overview and to identify rather principled approaches. Based in these prerequisites, we have shown that gradient-based methods like (Benosman et al., 2012) are generally not stable in terms of their input feature estimation. The main reason is rooted in the potentially very small number of events generated at a single location (c.f. Figure 1). Based on these investigations we have further shown that the numerical approximation of the gradients, like in Benosman et al. (2012), has methodological deficiencies that may lead to inconclusive motion estimates. On formal grounds, we have demonstrated that a gradient-based motion detection and integration scheme, using the scheme of Lucas and Kanade (1981), can be utilized to numerically estimate second-order spatio-temporal derivatives on a function that represents the temporal derivative of the luminance distribution. This requires to employ proper numerical difference schemes which also demonstrates the disadvantage of increased noise sensitivity (Section 2.1.3).

In contrast, methods exploiting the local structure of the cloud of events are more robust in general. Here, we compared different approaches. First, we reviewed methods fitting an oriented plane to the event cloud. We derived equations which demonstrate that the orientation parameters of the plane directly encode the velocity [see Equation (18)]. The benefit of such an approach against the above-mentioned numerical derivative scheme is that it works even in the case of only a few generated events. Of course, the goodness of fit depends on the size of the spatio-temporal neighborhood. However, if we consider a neighborhood that is too small then the plane fit may eventually become arbitrary and thus instable. If the neighborhood is too large then the chances increase that the event cloud contains structure that is not well approximated by a local plane. This also applies to the case of multiple motions, such as in the case of, e.g., occlusions due to opposite motions, limp motion in articulations, or in case of transparent motion stimuli.

Based on these insights we suggest a novel filter that samples the event-cloud along different spatio-temporal orientations. Its construction “reverses” the singular-value decomposition conducted of V1 receptive fields to construct direction-selective cells with spatio-temporally inseparable receptive fields (De Valois and Cottaris, 1998; De Valois et al., 2000). The conducted theoretical analysis allows to realize a spatio-temporally selective filter bank. Our investigation is similar to Escobar et al. (2009) who seek to specify the spatio-temporal selectivity. In contrast, our mechanism is directly derived from physiological findings. Perhaps the most similar scheme in comparison to our model is the one proposed by Adelson and Bergen (1985) which also suggests to derive spatio-temporally selective kernels by superposing different receptive fields. In their work, a spatial quadrature pair and two bi-phasic temporal kernels (in contrast to the mono- and bi-phasic kernels employed in our work) are combined (Adelson and Bergen, 1985) (compare also the review Emerson et al., 1992 and Borst and Egelhaaf, 1993). This scheme was motivated to resemble the spatio-temporal correlation scheme for motion detection (Hassenstein and Reichardt, 1956; Reichardt, 1957). In contrast to their approach, we rely upon the superposition of space-time separable filters with *out-of-phase* temporal modulation filter-responses. In addition to the main analysis, our test applications of the model implementation successfully demonstrate the functionality of such initial filtering for motion detection from spatio-temporal event clouds.

Compared to plane-fitting models (as suggested by, e.g., Benosman et al., 2014) we have shown that our model has the advantage that it can encode multiple motion directions at a single location, such as, e.g., (semi-) transparent motion (Figure 10; compare, e.g., Snowden et al., 1991; Treue et al., 2000; see e.g., Raudies and Neumann, 2010; Raudies et al., 2011 for a detailed discussion of motion transparency computation).

### 4.2. Non-linear response normalization by divisive inhibition

In order to account for non-linearities in the response properties of cortical cells (Carandini et al., 1997) several models have been proposed to arrive at a neural circuit to define canonical computational mechanism (e.g., Kouh and Poggio, 2008; Carandini and Heeger, 2012). These and other models employ a mechanism of divisive inhibition of the surround activity (also used here) that has been suggested to explain findings ranging from gain control (Ayaz and Chance, 2009; Louie et al., 2011) over attention effects (Reynolds and Heeger, 2009; Lee and Maunsell, 2009; Montijn et al., 2012) to normalization in multi-sensory integration (Ohshiro et al., 2011). Tsui et al. (2010) have demonstrated that cells in the motion-sensitive area MT can properly respond to motion directions even for tilted bars although the normal flow directions signaled by component sensitive V1 cells should bias the motion selectivity in a direction orthogonal to the tilt direction. These authors suggest a divisive normalization that operates upon the static filters of oriented contrast filtering *before* the separate temporal filter. Such a scheme is rather implausible mechanistically. We therefore developed a scheme that employs the pool normalization after the stage of spatio-temporal event-input filtering (c.f. Brosch and Neumann, 2014b). The simulation results using oriented bar stimuli further confirm findings of Guo et al. (2006) in which enhanced responses were shown at the bar ends while the responses along the extended boundary of the bar are significantly reduced [consistent with earlier investigations Bolz and Gilbert, 1986]. While Escobar et al. (2008) showed that such a reduction of uncertainty can be achieved by using subtractive surround inhibition the proposal by Bayerl and Neumann (2004) suggests that feedback can reduce such redundant aperture responses. Taken together, the proposed model not only demonstrates that response normalization of initial motion detection successfully operates for event-based representations but suggests a reasonably simple account for the recent experimental observations (Tsui et al., 2010) using lateral interactions.

Based on statistical investigations, a decorrelation of the responses of a group of cells into rather independent components has been suggested in Lyu and Simoncelli (2008a, 2009a), dubbed *radial Gaussianization* to account for the broadening of the tuning curves. Since we showed certain similarities but also deviations from the model proposed here, we employed an information theoretic measure which confirms that the normalization scheme decorrelates input representations by decreasing the multi-information even without special parameter learning from a test set (Studený and Vejnarová, 1998; Lyu and Simoncelli, 2009a,b). This might be beneficial in light of coding principles (to support a sparse coding mechanism, Olshausen and Field, 2004) and to better deal with the variability of the overall motion stimulus configuration. For example, most model mechanisms have been employed by assuming (implicitly or explicitly) that the motion can be approximated locally by translatory motion. However, for cases of rotations, the intersection-of-constraints mechanism (Adelson and Movshon, 1982) fails as there is no common point of intersection from local estimates (Caplovitz et al., 2006). We suggest that such a stage of normalization in real-world motions reduces the response to ambiguous parts of a stimulus, like the center of an extended contrast. At the same time due to the reduced mutual dependency of individual responses in a population the rotation components can be combined into a more global configuration more easily. This is exemplified by demonstrating the effective pushing of the motion response histogram toward the true motion direction (Figure 9) similar to Tsui et al. (2010) (see Pack and Born, 2001 for a discussion of an account to solve the aperture problem in area MT).

### 4.3. Summary

Motion estimation from the output of an asynchronous event-based vision sensor requires adapted methods. Here, we conducted for the first time a theoretical investigation that systematically categorizes event-based flow estimation models with respect to their underlying methods, namely gradient-based methods and algorithms exploiting the locally approximated plane-like structure of the cloud of events. In addition to analyzing existing gradient-based methods inconsistently mixing first and second order derivatives we proposed a novel consistent gradient-based algorithm. Even further, we showed that gradient-based methods in general suffer from strong noise originating from the limited number of events occurring at a single location. Methods exploiting the local plane-like shape of the event-cloud, on the other hand, were shown to be suitable for motion originating from a single object. In addition, we derived an explicit formula to derive the velocity from the parameters of the plane. For filter-based approaches, we proposed and analyzed a novel biologically inspired algorithm and demonstrated that it can also deal with motion transparency, i.e., it can represent different motion directions at a single location. Finally, we analyzed the impact of a stage of response normalization. We demonstrated that it is applicable to flow originating from event-based vision sensors, that it reduces motion ambiguity, and that it improves statistical independence of motion responses. All the theoretical findings were underpinned by simulation results which confirm that the model robustly estimates flow from event-based vision sensors.

## Author contributions

Designing the models/experiments: TB, ST, and HN. Mathematical and theoretical analysis: TB and HN. Spatio-temporal filter-analysis: TB. Experimental investigations: ST. Manuscript preparation: TB, ST, and HN.

### Conflict of interest statement

The authors declare that the research was conducted in the absence of any commercial or financial relationships that could be construed as a potential conflict of interest.
